# Perceived Benefits, Harms, and Views About How to Share Data Responsibly: A Qualitative Study of Experiences With and Attitudes Toward Data Sharing Among Research Staff and Community Representatives in Thailand

**DOI:** 10.1177/1556264615592388

**Published:** 2015-07

**Authors:** Phaik Yeong Cheah, Decha Tangseefa, Aimatcha Somsaman, Tri Chunsuttiwat, François Nosten, Nicholas P. J. Day, Susan Bull, Michael Parker

**Affiliations:** 1 Mahidol University, Bangkok, Thailand; 2 University of Oxford, UK; 3 Thammasat University, Thailand

**Keywords:** Thailand, data sharing, consent, collaboration, research ethics

## Abstract

The Thailand Major Overseas Programme coordinates large multi-center studies in tropical medicine and generates vast amounts of data. As the data sharing movement gains momentum, we wanted to understand attitudes and experiences of relevant stakeholders about what constitutes good data sharing practice. We conducted 15 interviews and three focus groups discussions involving 25 participants and found that they generally saw data sharing as something positive. Data sharing was viewed as a means to contribute to scientific progress and lead to better quality analysis, better use of resources, greater accountability, and more outputs. However, there were also important reservations including potential harms to research participants, their communities, and the researchers themselves. Given these concerns, several areas for discussion were identified: data standardization, appropriate consent models, and governance.

The Thailand Major Overseas Programme or Mahidol Oxford Tropical Medicine Research Unit was established in 1979 as a research collaboration between Mahidol University in Thailand and University of Oxford in the United Kingdom. At Mahidol University, the Programme is part of the international department of the Faculty of Tropical Medicine. The main office and laboratories are located within the Faculty of Tropical Medicine in Bangkok, Thailand, but research is conducted in many different locations both in Southeast Asia and more widely in Africa, South Asia, and Europe. The Programme attracts internationally renowned Thai and foreign researchers from all over the world. At any one time, the Programme has around 60 to 70 active clinical studies on malaria and other neglected diseases such as melioidosis and unexplained fevers.

In recent years, the Programme has coordinated some of the largest international studies involving many sites in low-income and hard-to-reach settings (Ashley et al., 2014; Dondorp et al., 2010; Onyamboko et al., 2014; South East Asian Quinine Artesunate Malaria Trial Group [SEAQUAMAT], 2005). In such studies, Bangkok, with the support of the Clinical Trials Support Group, has acted as the hub for study management and coordination. Within the Clinical Trials Support Group, there are seven professional data managers who perform data management using commercial US FDA CFR Part 11 compliant data management software, MACRO EDC (www.infermed.com) and OpenClinica (www.openclinica.com). The majority of the studies conducted by staff in the Programme are sponsored by the University of Oxford. With 700 to 800 personnel distributed across its collaborative research network, the Programme generates vast amounts of research data.

It has been our policy for many years to support sharing of data across collaborative research networks to maximize their utility. However, there is increasing support from research funders, regulatory agencies, and journals for sharing individual-level data from genomic, medical, and public health research beyond research collaborations (European Medicines Agency, 2014; Godlee & Groves, 2012; Harris, 2011; Medical Research Council, 2011; National Institutes of Health, 2003; Nisen & Rockhold, 2013; PHRMA, 2013; Research Information Network, 2008; Toronto International Data Release Workshop et al., 2009; Walport & Brest, 2011; Wellcome Trust, 2009).

A number of potential advantages of sharing individual-level data from clinical and public health research have been identified in the literature. These include maximizing the utility of data, allowing verification of research results, and minimizing the burdens and costs of unnecessary duplication of research (Doshi, Goodman, & Ioannidis, 2013; Hrynaszkiewicz & Altman, 2009; Manju & Buckley, 2012; Pisani, Whitworth, Zaba, & Abou-Zahr, 2010; Rani, Bekedam, & Buckley, 2011; Walport & Brest, 2011). In low- and middle-income settings, it may be particularly important to effectively share data to maximize its utility and enable timely responses to important public health issues such as resistance to antimalarial treatments (Langat et al., 2011). Many authors have called for data sharing to be carefully curated, to minimize potential harms including breaches of privacy, the publication of poor quality or biased secondary research, and insufficient acknowledgment of the contribution of researchers generating data sets (Mello et al., 2013; Pearce & Smith, 2011; Rabesandratana, 2013). In low- and middle-income settings, the need for data sharing policies and processes to promote equitable use of data, including the development of sustainable capacity to both share and analyze data sets, has been recognized (Sankoh & Ijsselmuiden, 2011; Toronto International Data Release Workshop et al., 2009; Walport & Brest, 2011).

To date, there have been very few empirically grounded accounts of data release policies for biomedical and public health research in low- and middle-income countries or the practical and ethical governance challenges raised in such settings (Bull, Roberts, & Parker, 2015). This article reports on the findings from one of five sites in an international qualitative study exploring the experiences and views of a range of stakeholders involved in medical and public health research in Asian and African settings (Denny, Silaigwana, Wassenaar, Bull, & Parker, 2015; Hate et al., 2015; Jao et al., 2015; Merson et al., 2015; Parker & Bull, 2015). As partners in this study, we wanted to understand the perceptions, experiences, and values of relevant stakeholders in Thailand about what they consider to constitute good data sharing practice. Findings from this study can assist us to develop policies and processes to share data in a way that mitigates potential harms and retains the trust and confidence of researchers, communities, and participants in our research.

## Method

### Context

The study was conducted in the Bangkok hub and at the Programme’s biggest research site, the Shoklo Malaria Research Unit in the Thai–Myanmar border town of Mae Sot. At the time of writing, there were approximately 170 staff members in Bangkok of whom three quarters were Thai. There were nearly 600 staff members at the Shoklo Malaria Research Unit, the majority of whom were from the “border community,” and a small number of expatriates.

Although many research studies are led from Bangkok, few clinical studies are conducted there as there are only a small number of patients who get malaria and other tropical diseases. The Shoklo Malaria Research Unit, by contrast, has been involved in providing health care and conducting operational research in the Burmese and Karen migrant population on the Thai–Myanmar border for nearly 30 years. The border zone has been an area of political conflict and occasional violence for many years. There is limited access to medical personnel and facilities on either side of the border, hence many migrants access our clinics and a non-governmental organization (NGO)–run clinic called the Mae Tao clinic (maetaoclinic.org). Our clinics are located on the Thai side of the border directly across the river from the Karen villages in Myanmar and within a large refugee camp.

### Participants

For this study, which aimed to gather experiences of and views about data sharing from a range of relevant stakeholders, we used a combination of purposive and convenience sampling. For research staff, we recruited staff working in the field, office, and laboratory, junior and senior researchers, those who direct research, and those who implement research. Unfortunately, only researchers and community representatives were able to be recruited to this study as we were required by the local ethics committee to exclude medical research participants.

We conducted a total of 15 interviews with research staff of which 13 were in Bangkok and 2 in Mae Sot. A focus group discussion with 3 members of the Clinical Trials Support Group was held in Bangkok, and a focus group with 7 community members was conducted in Mae Sot (see Table 1).

**Table 1.** Participant Composition.

	Bangkok		Mae Sot	
	Thai/Karen	Foreign	Thai/Karen	Foreign
Research staff	9	6	1	1
Community representatives	0	0	8	0
Total	9	6	9	1

All interviews and focus groups were conducted at a time and place convenient to the participants. Staff in Bangkok chose to be interviewed in the Programme office or laboratory. The interview with the community representative was conducted at her work place, and the focus group with the community representatives was conducted at the Shoklo Malaria Research Unit. The community representatives were affiliated with the Shoklo Malaria Research Unit where they had been hired as temporary community engagement staff. All participants signed a consent form prior to participation. They were specifically asked whether they would consent for their de-identified transcripts to be shared within the collaborative research team as well as outside the research team. All who were approached to be interviewed consented to data sharing within the research team and beyond, except for one participant who only consented to share the de-identified transcript with the research team.

All interviews and focus groups were conducted by P.Y.C., D.T., or A.S. using topic guides adapted from a template developed collaboratively with the partners from the other sites involved in this study (Parker & Bull, 2015) and are available upon request. For all interviews and focus groups, there was a lead interviewer (P.Y.C., D.T., or A.S.) and one or sometimes two note takers. Interviews and focus groups were audio recorded and then transcribed verbatim in the original language. Interviews and focus groups in Thai and Karen were translated into English. The transcripts were then checked for accuracy and de-identified. De-identification was performed by removing names of people mentioned, study titles, project names, organization names, and other information with the potential to identify the participant. Transcripts were then imported into NVivo 10 for coding and analysis. Initial descriptive coding was conducted by A.S., using a coding framework developed collaboratively with the other partners in the international study. The coding of half of the transcripts was reviewed by P.Y.C. and amended where necessary. In addition, a sample of transcripts was cross-coded by a researcher from another site. P.Y.C., D.T., A.S., and S.B. met to review the initial coding and to expand the codes to include inductive descriptive codes grounded in the data (Thomas, 2006). Findings from the descriptive coding were discussed with the international research group during an analysis meeting in July 2014. At that meeting, a thematic framework and analyses charts were developed for the subsequent analysis (Gale, Heath, Cameron, Rashid, & Redwood, 2013). Data from the study are available, please contact the corresponding author for details.

### Ethics Review

This study was approved by the Oxford Tropical Research Ethics Committee as part of the collaborative study (OxTREC 1051-13) and the Faculty of Tropical Medicine Ethics Committee (MUTM2013-052-01), Mahidol University, Thailand, for the conduct of the study in Thailand.

## Results

Our participants identified a number of potential advantages to data sharing and were in general positive about it. However, they also described a range of possible harms and worries about the sharing of data. When asked, participants were able to suggest various possible measures with the potential to address or protect against negative aspects of sharing data from low-income settings. As expected, experiences of data sharing among participants varied considerably. Most researchers had limited experience of sharing data, but had shared data with collaborators and international organizations including the World Health Organisation, the Worldwide Antimalarial Network (http://www.wwarn.org), the U.S. Food Drug Authority, the Walter Reed Army Institute of Research for drug development, and national authorities including ministries of health and national malaria control programs.

In what follows, we begin by presenting some data on the personal experiences of participants before going on to present their views on the potential benefits and harms of data sharing and the measures they propose for addressing these harms.

### Personal Experience

Although most researchers interviewed perceived benefits of data sharing, most had little personal experience of sharing or accessing data beyond their experience of sharing data within research collaborations. Researchers’ experiences were largely limited to sharing data with collaborators they knew, trusted, and had successfully worked with in the past or with those who were in “close proximity” to the Programme. Some interviewees suggested that this was partially a decision they had made rather than a lack of opportunity to share outside their research group.

My approach to sharing data is, depends on, and is very much guided by the proximity with the organization, or the person or the group we share the data with. The proximity in terms of collaboration, if it is a very close group that we have collaboration and we have an understanding . . . we can trust each other and we can work together and we have a common goal, then, usually I don’t have problem with sharing data, any type of data. But if it is a more distant group or competitor then we don’t share data. (TH-SR-I-15, Senior Researcher, male)

Some senior researchers had, however, had experience of making a limited data set of raw data open access following publication of the analysis in journals. Some reported good experiences, but others reported concerns including not being offered co-authorship or even acknowledged in publications where this was expected.

We had some studies that we sent our dataset to the Ministry of Health in X [country in South East Asia] and they gave it Y [Western agency] . . . Y used this data and didn’t mention us at all . . . our contribution to the data that we had done. So they just credited . . . they just gave one name for that Ministry of Health and the rest are all the Y names. So we think that this is unfair . . . well I saw the manuscript and the few of us just wrote to them and say look I think there is a mistake because I know this data that you published, I know how it was collected, I know all the people are involved in it and I think you should give them credit. (TH-SR-I-08, Senior Researcher, male)

Most of the researchers with personal experiences of data sharing were relatively senior; junior researchers and other research staff had more limited experience with data sharing. Against this background of varied experience, the following sections outline the views of participants about the benefits and harms of data sharing and their suggestions for good data governance.

## Benefits of Data Sharing

All of the researchers we interviewed regarded data sharing in a broadly positive light. They recognized the potential advantages of data sharing and were positive about the opportunity for sharing data they had already gathered. Participants outlined a range of potential benefits. These fell into five broad themes: promoting scientific progress, better analysis and bigger data sets, greater accountability and transparency, better use of resources, and the potential for benefits to accrue to the researcher and research group.

### Promoting scientific progress

All of those interviewed saw the potential for data sharing to contribute to scientific progress and ultimately to patient benefit if done in the correct way. The researchers we spoke to were unanimous that if conditions were right, data should be shared to maximize their benefit to the public and ultimately improve lives. This suggests that support for data sharing was grounded on recognition of the importance of beneficence. For some participants, this was connected to a reluctance, or at least a wariness, to share with commercial companies but others thought that as long as the data were going to be used in the public interest, for example, to benefit public health, then ultimately, the data should be shared.

The information should be shared because who knows whether the information you provide is going to help with the development of a new drug that you know is going to improve the lives of many. So I think we have to overcome this obstacle or this perception that we are willing to share with our peers but we’re not willing to share with those who have a lot of money. So I think we have to overcome that, and it’s really, goes back to the altruism . . . the focus is on the patient rather than on the Nobel Prize. (TH-SR-I-03, Senior Researcher, male)

Some researchers gave additional ethical arguments for data sharing in particular cases, such as where data sets are particularly valuable or in the context of emergencies. As an illustration of a particularly valuable data set, one researcher highlighted the fact that there were strong ethical reasons for sharing data sets where the collection of valuable data would be impossible or unethical to repeat. The researcher illustrated this argument by using the example of a large randomized controlled clinical trial involving more than 5,000 African children with severe malaria with mortality as the endpoint (Dondorp et al., 2010). The researcher argued that given the time and resources required to conduct such a large study, it is unlikely that others will independently repeat a similar experiment. In addition, for some of the diseases studied, for example, severe malaria in children, due to the global decline in the number of fatal severe malaria cases, it would be impossible to repeat the study. In situations such as this, where data collection cannot be repeated and the data are of great value, the researcher argued that it is imperative that the existing data set be reused to maximize the value of the study and to do justice to patients who have altruistically participated in these studies.

The researcher in the quote below argued for data sharing even before publication in the case of emergencies and public health issues, such as artemisinin resistance in malaria, adverse drug reactions, and outbreaks such as the recent Ebola outbreak:

The data on resistant malaria, there is clearly a very important public interest . . . policies will depend on it and the whole containment effort for malaria . . . might be influenced by data . . . research groups should share data even before publication because policies might be changed because of that. (TH-SR-I-07, Senior Researcher, male)

### Better analyses and larger data sets

Relating to the theme of “promoting scientific progress,” participants argued that data should be shared because of the potential value of complementary approaches to data analysis. Data sharing enabled data sets to be analyzed using alternative methods, allowing researchers with different skill sets, backgrounds, and interests to ask different questions, and thus to come up with new or additional results. In addition to the potential value of new methods, the merging of small data sets was also thought to have the potential to enable more powerful statistical analyses to be performed so that, for example, much smaller differences between drug effects could be seen.

As an illustration of this, one researcher we interviewed described how a recent pooled analysis of an antimalarial drug in children revealed that there was under-dosing, and enabled the evidence-based development of a new dosing recommendation (WorldWide Antimalarial Resistance Network DP Study Group, 2013). Another potential methodological advantage of data sharing is the greater scope it offers for imputation and better prediction of gaps in the data. Some participants also argued that data shared with other researchers meant that these data could be verified and built on, allowing any errors in data analyses to be detected. In their view, verification was essential to an open dialogue about research results.

### Greater accountability and transparency

For most participants, in addition to its scientific value, data sharing was viewed as a way to improve scientific accountability and transparency. The view of these participants was that if data sharing were envisaged at the outset of data collection, this would minimize fraud or even serve as a deterrent because the wider scientific community would have the opportunity to reanalyze the data and confirm the results. It was also recognized that the accountability and transparency data sharing brings also have important scientific benefits because independent verification is a time-tested strategy for quality control.

The data should be shared because what we have done . . . whether it is good or bad, or it is right or it is wrong . . . if we share, we can . . . improve our team or the way we work . . . we will improve ourselves. (TH-JR-G-02, Community Representative, male)

### Better use of resources

Another benefit of data sharing raised by participants was its potential to limit the need for duplication, except in situations in which replication is necessary to confirm results. This would mean that limited resources, including researcher time and research funds, could be used for things other than the collection of pre-existing data. All agreed that the use of data already collected could be and should be improved. One interviewee thought that before any researcher or research group embarked on the collection of new data, they should be required to find out whether there is an existing data set that could be used to answer their question.

It saves money, saves time, saves human resource . . . in case if we can collect data, just collect data to fit in our study . . . to compare to get the results. (TH-JR-I-06, Junior Researcher/Research Nurse, female)

Many researchers were of the opinion that receiving data from another group was an efficient way to perform research, provided the data meet certain quality standards. Secondary use of existing data is normally essential for students, early career researchers, and researchers who do not have a lot of funding. In addition, it is a useful resource to be used to aid planning of future studies that involve collection of new data.

### Benefits for researchers and research groups

Finally, many senior researchers saw the sharing of their data as not only contributing to scientific progress but also helping them in fostering collaborations and improving their research profile. In addition, the data collector could reap important benefits from sharing data with groups that have different expertise. As mentioned above, scientific benefits could be gained if data were pooled from different groups and the researchers from different groups collectively conducted analyses. Apart from its scientific benefits, such collaboration could lead to new results and publications, which could in turn help the careers of those who share.

Data sharing is useful when it meets the needs of the future. If we start collecting the data and we think that it might be used in the future, it’s good. However, if it’s a kind of data that can’t be shared then it’s useless. Therefore, we should think about data sharing from the beginning of the study, to think about what kind of data we might want to use in the future. We should structure what we want to collect beforehand, before the study starts, to make sure that data you collect can be used now and also in the future. If the data that you collect can’t be shared it is useless even to collect now. (TH-CTSG-G-01-R1, Clinical Trials Coordinator, female)

It was recognized, however, that benefits for individual researchers or research teams would only materialize if attribution was appropriately granted through authorship, acknowledgment, or future funding.

## Concerns and Harms

Although the researchers we interviewed were largely in favor of data sharing and identified the range of benefits outlined above, they also worried about the potential harms of, and barriers to, successful data sharing. There was a strong sense among researchers that the benefits of data sharing would only be fully realized if measures were put in place to address these potential harms and barriers. The concerns identified by participants clustered around three broad areas: the potential for harms to patients and communities, the potential for harms to researchers and research groups, and the resources required for data sharing.

### Potential harms to patients and their communities

One important worry that those interviewed had was about the potential harms that might arise out of the identification of participants/patients as a result of the linking of different data sets. This reservation seemed to be felt most strongly in relation to clinical research data. There were concerns that sharing data might compromise patient confidentiality, which could cause harm. This was considered most likely when sensitive data were shared outside the original research team. It was believed that these risks to study participants could not always be fully mitigated by the de-identification of individual data.

Beyond the individual, participants also worried that sensitive data might lead to stigmatization of the whole community. Important examples included HIV/AIDS, mental illnesses, and susceptibility to malaria. Even where identifiable information about patients is routinely removed from data sets, publication of findings might result in whole communities being stigmatized or discriminated against. This could result in loss of insurance coverage or ineligibility for certain job opportunities.

But if you collect some demographic data, for example, some initial or some region, it is possible that they can track back. Even though you didn’t put the name, the 13-digit ID, something else may allow you to track them, who they are . . . maybe the insurance company wants to get this information and want to know if this population in this region want to buy insurance, they may want to get it from you and they may know that these people got these diseases regularly. (TH-CTSG-G-01-R1, Clinical Trials Coordinator, female)

In addition to medical or health-related data, participants worried that non-medical data such as ethnicity, GPS locations, and patient addresses could be sensitive in some settings. Some of our interviewees were able to provide personal accounts of situations in which they had encountered individuals who did not want to reveal their identities or their addresses to health authorities for political, land ownership, housing, or other reasons.

For me, when we started Z [research project], my concern was when people wanted to start the GPS data collection. I personally felt insecure for people’s households. Right now they’re at peace, so they’re not going to shoot anyone, or hurt anyone but if they find out that . . . our location, maybe in the future they might use those information to hurt each other. So that was my concern, but I was explained [it was explained to me] later that it will be kept confidential. (TH-JR-G-02-R6, Community Representative, female)

In addition to worries about identification and its implications, some interviewees were concerned that disparate results obtained from re-analyses of the same data set by different groups could cause confusion and potential harm to patients. Against a broad background view that data sharing would be likely to improve science and ultimately the care of patients, there were concerns that a proliferation of poor quality analyses might make it difficult for those making treatment decisions to distinguish between valid and invalid findings. If researchers were irresponsible or if they had a specific agenda, they could trawl, in theory, through large data sets and find things they set out to look for. This practice, called “cherry picking,” would create misleading conclusions, leading to harms to future patients.

### Potential harms to researchers and research groups

As was highlighted earlier in this article, some researchers had had personal experiences of not being appropriately acknowledged by those who had used data produced by them. This led to concerns that the conduct of secondary research could deprive original investigators of an appropriate return on their investment of time and expertise. Original researchers would be at risk of being scooped if others came up with groundbreaking analyses using their data set. This would be particularly problematic where data generated were not acknowledged appropriately. In addition to their short-term impact, such harms would also jeopardize the original researchers’ future funding opportunities and career advancement. These concerns were particularly relevant when sharing large high-profile data sets where a research group may have historically had the prestige of being identified with that data set. In such cases, data sharing might lessen potential opportunities for collaboration or attracting additional research funding based on their ownership of the data set.

We are all in the business, profit and business. So, researchers, they don’t produce anything that you can sell. I am not making mobile phones or I am not making plastic ware. I am making data and knowledge. I cannot sell them. The only thing I can do is produce the result that will convince the sponsor to give me money to continue to produce results . . . so, because we are now living in the world of the economic model like that, if people are using my work to make money for themselves, because if they use the data they publish paper, their rank goes higher, they get more funding and they get money, not me. (TH-SR-I-15, Senior Researcher, male)

Finally, another potential source of harm to research was seen to arise out of the possibility that re-analyses, possibly of poor quality, might come to different conclusions or in some cases, identify mistakes in the data or the original data analyses. In such cases, original researchers and their institutions would be at risk of reputational damage.

### Demands on resources

In addition to the potential harms to patients and to researchers, most of those interviewed also highlighted the fact that for data to be shared, and for the benefits of data sharing to be realized, significant time, effort, and resources need to be invested into ensuring that the high-quality data sets are shared in an understandable way. Data can only be used to their full potential by other researchers if they are well curated. Participants believed that this required much more than simply ensuring that data were organized and well-managed. For the value of research data to be fully realized, any potential user would also need to understand exactly how the original research was carried out, what its purpose was, and what the data meant. This means that for data to be useful to the recipient, the data creator would need to provide sufficient information on the objectives and methodology of the research; explain the data collection methods used; and describe the meanings of variables and codes used and any derivation, transformations, or data cleaning. Our participants were strongly of the view that sharing bad or poorly curated data was not only useless but also potentially wasteful or harmful.

It is unethical to have poorly collected data and put it out there because it can mislead, it doesn’t do justice to participants who signed up for the study. So it’s incumbent to provide adequate data management. (TH-SR-I-12, Senior Researcher, male)

This means that effective data sharing comes as a cost. Participants noted that data management is an expensive business. It requires experts who are skilled in data collection, data validation, standardization of variables, and tabulation. They added that these were skills that were rare in low-income settings, particularly in academia, so there is often little or no capacity for data management in developing countries. In addition, data management software is expensive and only few academic groups can afford it. Participants argued that given the resources required to create and maintain a data set that is usable by other groups, and the opportunity costs of spending time on this, there is little incentive for researchers in low-income settings to prioritize curating data for sharing over conducting new studies. As a consequence, in general, in low-income settings, unless the research study is commercially funded or the research group has its own data management team, data are generally not curated to the standard required for sharing.

Although most of those interviewed agreed that good quality data were a prerequisite for effective sharing, one researcher believed that as long as the data set was large enough, errors would be relatively small and would not affect the overall conclusions. He argued that it is important to acknowledge that no data set is perfect and as long the original researchers declare what quality control measures have been undertaken, then the data set can be of potential value.

## Suggestions for Best Practices in Data Sharing

In the previous two sections, we have outlined the views of participants about the potential benefits and harms of data sharing. All participants saw the advantages of sharing data from low-income settings and were interested in exploring ways in which the potential harms might be addressed and appropriate protections put in place. During their interviews, participants suggested a number of ways in which the harms and worries they have might be addressed. These suggested solutions fell into three broad groups: ensuring that data were of good quality, high standards of consent, and better data governance.

### Resources and capacity to ensure good quality data

Many of the researchers we interviewed were of the opinion that good quality data were a prerequisite for effective and useful data sharing. They took the view that there is an urgent need for capacity building around data-curation, management, and analysis in low-income settings, and that this needed to be addressed before seriously considering data sharing. Participants emphasized the importance of establishing high standards of good practice if potential benefits of data sharing are to be realized. Most researchers and data managers believed that there was a need for a “quality guarantee,” and in addition to good quality data, many emphasized the importance of good quality supporting documentation including the study protocol, case report forms, data management plan, and data dictionaries. Some felt that effective data sharing was still possible as long as the nature and extent (and limitations) of quality checks in the data were disclosed. However, all agreed that the most effective data sharing could only take place in the context of well-managed and curated data sets. As described in the section above, all participants were clear that curating high-quality data has resource implications, and all saw the need for additional funding and capacity building as prerequisites for effective global health data sharing.

### Consent

All of those interviewed were concerned to ensure that the interests of research participants and communities were protected, and most saw effective valid consent as a key element in ensuring that this was the case. Different models of consent were discussed in the interviews and focus groups discussions; however, no agreement was reached on what would be the best approach. It was agreed by all respondents that research participants should have some say about what happens to their data. How this should be done in practice was more complicated. Many of those interviewed were advocates of obtaining broad consent for future use of data; however, some saw that as a “burden” to participants because it is difficult to explain to participants the wide range of possible future uses for their data and the possible types of requesters. It was felt that broad consent could only be valid if there was some clarity at the time of consent that the data would be reused, about the kinds of people or institutions they would be shared with, and about how, in broad terms, they would be likely to be used. More explicit consent should be sought where the research questions were significantly different from those envisaged at the time of data collection.

Interviewees highlighted the fact that many studies conducted by researchers in the past had not obtained consent for data sharing, broad or otherwise, as data sharing was not anticipated when data were collected. When asked whether these research participants should be approached to be re-consented, some felt that if the research would benefit the community participants were drawn from, then the data could be used without additional consent. Other researchers felt that if the research has social value, not necessarily benefiting the same community but of some benefit, the data could be used without additional consent. However, not everyone agreed with this view, as the data were not considered to be the investigators’ to give. It was their view that research participants should be told that their data would be shared.

But the original data, you get it from subjects you didn’t collect data from investigator; you collect the subjects’ data . . . You have to tell them in the consent because you got data from investigators but it’s not investigators’ data, it’s subjects’ data. (TH-CTSG-G-01-R1, Clinical Trials Coordinator, female)

During a focus group, this comment led to an interesting discussion about the arguments in favor of and against seeking broad consent and led ultimately to a shared view that although valid consent is important in all studies, different approaches would be required in different contexts.

Right, but you have to find the participants again and reconsent and it’s complicated, especially if the research is done in the remote area. Yeah I mean I think it depends on the situation, if the research is in the remote area then probably broad consent is more appropriate, whereas if the research is done in an urban hospital, for example where the patients can easily be tracked then the explicit one might be more appropriate . . . it depends on how to track the patients down. (TH-DM-I-11, Data Manager, female)

Interviewees discussed the practical difficulties of re-consenting such as inability to trace participants either because they live in remote areas or because data had already been delinked from patient identifiers. Some interviewees felt that if the data are not “sensitive” such as parasite counts for a malaria study, then they could be shared with other researchers without re-consenting, irrespective of the type of research questions secondary research would address.

The requirement for explicit consent was emphasized in the context of research involving commercial partners or where there was the potential for commercial profit. If the data were used in studies where results could yield commercial gains, then respondents thought that explicit consent should be sought from participants on a case-by-case basis.

### Governance

Given the potential harms that data sharing might bring about, participants felt that for data sharing to be successful, it needs to be appropriately governed, managed, and funded. The discussion of data governance focused primarily on the strengths and weaknesses of open versus managed access approaches to data sharing.

#### Open access

Very few researchers were in favor of having the entire data set, including unpublished data, publicly available without any controls. This was primarily because of concerns about the potential harms described above. However, one interviewee said that if a published data set could be considered a publication in its own right and granted the same prestige as an academic paper, then that would address many of the concerns about potential harms to researchers and research groups. Almost all researchers were in favor of making data on which publications were based publicly accessible. This had already been practiced to a certain degree as it is mandated by some journals. However, importantly, this requirement normally only refers to a partial data set from which published findings are derived rather than to the data set as a whole.

#### Managed access

The vast majority of those interviewed thought that given the potential harms of open access to data sets, a managed approach in which a governance committee or trusted gatekeeper vetted requests for access to data and ensured appropriate attributions would be preferable.

The gatekeeper should be someone who is qualified, and have time to deal with this. Most important thing that I concern is that investigators don’t have time, especially they will make things quick and let go of everything, just like snap finger. I was thinking about someone who is actually dedicated to do this task . . . you have to make them promise, maybe they may have to make some promise like you will not distort the data, you must not change the story, do things honestly and straightforward. (TH-CTSG-G-01-R1, Clinical Trials Coordinator, female)

Researchers thought that the requester should ideally have the proposed analysis pre-specified, formalized as a detailed study protocol, approved by relevant ethics committees, and registered on a suitable website. This process would ensure that there is transparency and rigorous peer review. It would also address some of the potential harms raised by the interviewees such as “cherry picking” and worries about the widespread dissemination of poor quality analyses. In addition, the proposal could be scrutinized for its potential benefits. This would address contrasting views about beneficiaries. The majority of participants said that data should be shared as long as the data shared are for public good. However, one researcher had strong opinion that data sharing must directly benefit the community that contributed the data.

Despite the majority of interviewees being in favor of a managed approach to data access, they did recognize some potential limitations of this approach. First, it was acknowledged that this would require resources. A gatekeeper or a committee could be costly, and the expenses and staff required would need to be identified in the project budget at the outset and funding provided by the research funder. It would also be necessary to apportion time and effort beyond the life of the initial study, that is, data sharing might potentially go on for a significant period beyond the end of the initial research project.

Another potential disadvantage of a managed approach is that it might deter applicants as potential data users might be put off by the application requirements. It might be especially difficult for those unfamiliar with the specific requirements for data access, and this might disproportionately affect people from low-income countries.

The thing that I would predict might be the biggest obstacle is xenophobia you know if you got a big datasets built up by someone like A (American funder), it’s likely that it’s gonna be very easy to access as a American researcher and really difficult to access as anybody who’s not an American. Equally if you are B (U.K. funder), you might be eyeing to access it from England, Africa, Southeast Asia, sort of people that the B deals but if you sort of put your head out above the parapet in Japan and say yeah, can I order your data, you might not be familiar with the sort of the processes that enable you get pass the hurdle. (TH-SR-I-01, Senior Researcher, male)

## Discussion

As discussed above, participants were unanimously in favor of sharing individual-level data in principle; however, many also had important reservations. Potential advantages of data sharing echoed those discussed in the literature to date and included promoting scientific progress, improving accountability and transparency in research, and maximizing utility of data sets (Bull, Cheah et al., 2015). Reservations about data sharing were underpinned by concerns and perceived potential harms clustered around three broad themes: potential harms to patients and communities, potential harms to researchers and research groups, and concerns about the availability of resources required for effective data sharing. Interestingly, although these concerns have been discussed in relation to sharing individual-level data in a variety of contexts (Eichler, Petavy, Pignatti, & Rasi, 2013; Langat et al., 2011; Manju & Buckley, 2012; Pearce & Smith, 2011; Rani et al., 2011), interviewees in this study primarily raised them in relation to the sharing of data beyond existing or previous collaborators, that is, with the wider scientific community. Some interviewees had extensive experience of sharing data with their collaborators in large-scale studies and were comfortable with and accustomed to merging data sets and having reciprocal data sharing arrangements with such groups. However, many of those interviewed had concerns about sharing data with people they do not know or have not worked with. This is partly because of their limited experience of such sharing, partly because of the concerns outlined above, and partly because some of those interviewed had had personal experiences of bad practices, such as inappropriate attribution.

In this article, we have outlined suggestions made by participants about how potential harms of data sharing might be mitigated to make data sharing with the wider scientific community a reality. These include addressing data quality, developing a model of good consent practice, and establishing an effective and trusted approach to data governance. Although open access approaches might allow for maximum transparency and maximum utility, it was felt that potential harms to data subjects, data collectors, and also to public trust might be best mediated through the adoption of a managed approach (Mello et al., 2013). It was clear that most participants favored a managed approach to data sharing rather than one that made data openly available, but interviewees believed that further discussion was needed about best to develop proportionate and effective governance. Concerns were raised that a controlled approach with the appearance of too many hurdles and too much bureaucracy may limit the ability of researchers from low- and middle-income settings to access data sets for secondary research. The question of how to govern data sharing to ensure fairness for all parties involved was equally challenging (Pisani et al., 2010; Walport & Brest, 2011).

It was also clear that a single approach may not be appropriate for all data sets, and what is required to ensure respect for data subjects and fairness between data sharers and data recipients is likely to need some case-by-case assessment (Greenhalgh, 2009; Pearce & Smith, 2011). It was acknowledged that there are already some examples of data repositories such as iSHARE2 (http://www.indepth-ishare.org/index.php/home) and MalariaGEN (Parker et al., 2009), which could be learned from.

Researchers were clear that data they collected were not theirs to give away and were of the view that this generated important obligations and duties. It was felt by most respondents that data belong to the research participant and not the researcher. Nonetheless, this did not necessarily mean that data should not be shared, even sometimes without explicit specific consent. It was felt, for example, that respect for a volunteer’s altruism meant that the utility of the data should be maximized, and in many cases, this required that such data should be shared with the wider scientific community. It was agreed that consent is one of the most important ways to address the issue of respect, but the right model of consent, whether broad consent, explicit consent, or re-consent, was difficult to prescribe in the abstract without a specific data set, a specific population, a specific requester, or a proposed secondary analysis in mind.

## Best Practice

One of the strongest themes emerging from our data was the need for further work to develop key principles of good data sharing practice capable of creating the sustainable trust and confidence required for the effective sharing of data for the benefit of participants (Bull, Cheah et al., 2015). A second and related theme was the importance of ensuring the availability of sufficient resources and capacity building.

It was striking in our context that there was a qualitative difference in attitudes toward sharing data with collaborators and sharing with the wider scientific community. This suggested one possible step that might be taken to maximize the benefits of data sharing would be to create strategic collaborations where data sharing would be built in from the outset by design or through collaborations with secondary users. An advantage of collaborations is that all potential users would have a better understanding of the data. To create such collaborations, researchers and relevant stakeholders, including international and national bodies could collaborate in designing the study, including endpoints and how and where data would be collected, curated, and tabulated for maximum use by all interested parties. In such a context, the data generated would be the joint responsibility of the members of the, potentially very large, research team.

## Research Agenda

The data collected in this study represent the experiences and views of a small number of researchers of the Thailand Major Overseas Programme in Bangkok and Mae Sot, and community members on the Thai–Myanmar border. We were required by our local ethics committee to exclude research participants from this study because research participants would not have heard of the concept of data sharing, and it would be too abstract for them to engage with. This suggests a need for further research into best practices with community members and research participants—potentially using a deliberative approach to introduce the topic to those who do have not previous experience of it (Haga & O’Daniel, 2011; Kim, Wall, Stanczyk, & De Vries, 2009; Marsh, Kamuya, Rowa, Gikonyo, & Molyneux, 2008; Molster et al., 2013). In addition, due to the limitations of time and funding, the study was only conducted at two locations, our Bangkok administrative hub and Mae Sot. Although the study identified a broad range of issues pertinent to an international research group in Thailand, we would have liked to obtain views of stakeholders at our other research sites, such as Cambodia and the Democratic Republic of Congo, where the political structure and research environment are significantly different from that of Thailand. This suggests a second avenue of future research.

Although the primary focus of this study has been on data sharing, it was clear that for many of those we spoke to, concerns about data sharing were closely interwoven with concerns about the sharing of biological samples. There is a growing interest in biobanking to facilitate researchers’ access to high-quality samples as well as to research data and medical records. To date, there are no established guidelines on biobanking in Thailand or in the other countries in Southeast Asia and Africa with which we collaborate. A study that aims to explore perceptions of and attitudes toward sample sharing and biobanking would complement the results of this study.

## Educational Implications

There is a need for capacity building in a number of key areas if effective, ethical, and sustainable data sharing is to become a reality. Many of the required skills are directly related to data storage and management and have been outlined above. It is important to review resources, policies, and processes developed to support data sharing in higher income settings and evaluate their value in low- and middle-income settings. To support this evaluation and the development of best practices in low- and middle-income settings further building of capacity in bioethics and social sciences is required. Such capacity building can facilitate the development of sustainable models of good practice, gather evidence about the effectiveness and acceptability of such models, and provide ethics support and advice to the research groups as they design and implement data sharing.
